# Yoke-Type Elasto-Magnetic Sensor-Based Tension Force Monitoring Method for Enhancement of Field Applicability

**DOI:** 10.3390/s24113369

**Published:** 2024-05-24

**Authors:** Ho-Jun Lee, Sae-Byeok Kyung, Ju-Won Kim

**Affiliations:** 1 Department of Nuclear · Energy System Engineering, Graduate School, Dongguk University, Gyeongju 38066, Republic of Korea; leehojun4504@gmail.com (H.-J.L.); saebyeok@dongguk.ac.kr (S.-B.K.); 2 Department of Safety Engineering, Dongguk University WISE, Gyeongju 38066, Republic of Korea

**Keywords:** tension force monitoring, yoke-type elasto-magnetic sensor, wireless communication, linear regression analysis

## Abstract

Tension members are key members that maintain stability and improve the strength of structures such as cable-stayed bridges, PSC structures, and slopes. Their application has recently been expanded to new fields such as mooring lines in subsea structures and aerospace fields. However, the tensile strength of the tension members can be abnormal owing to various risk factors that may lead to the collapse of the entire structure. Therefore, continuous tension monitoring is necessary to ensure structural safety. In this study, an improved elasto-magnetic (E/M) sensor was used to monitor tension force using a nondestructive method. General E/M sensors have limitations that make it difficult to apply them to operating tension members owing to their solenoid structure, which requires field winding. To overcome this problem, the magnetization part of the E/M sensor was improved to a yoke-type sensor, which was used in this study. For the development of the sensors, the numerical design and magnetization performance verification of the sensor were performed through eddy current solution-type simulations using ANSYS Maxwell. Using the manufactured yoke-type E/M sensor, the induced voltage signals according to the tension force of the specimen increasing from 0 to 10 tons at 1-ton intervals were repeatedly measured using DAQ with wireless communication. The measured signals were indexed using peak-to-peak value of induced voltages and used to analyze the signal change patterns as the tension increased. Finally, the analyzed results were compared with those of a solenoid-type E/M sensor to confirm the same pattern. Therefore, it was confirmed that the tension force of a tension member can be estimated using the proposed yoke-type E/M sensor. This is expected to become an effective tension monitoring technology through performance optimization and usability verification studies for each target tension member in the future.

## 1. Introduction

Tension members are important for maintaining the stability of a structure. These members are used as tensioning materials for steel wires and cables in structures such as bridges and tunnels [1]. Monitoring the tension in these tensioners is crucial because they are subjected to a variety of hazards, and any failure in their tension can lead to the failure of the entire structure. Recently, failures and mismanagement of maintenance due to the corrosion of tensioning materials led to the Morandi bridge collapse in Italy in 2018, which killed 43 people, and the Taiwan bridge collapse in 2019, which killed 5 people [2,3]. To prevent this, several precedents exist for measuring and monitoring the tension in tension materials. Studies have been conducted on estimating the cable tension of bridges using vision sensors on unmanned aerial vehicles [4], diagnosing defects in steel wire ropes using multichannel magnetic flux leakage (MFL) sensor-based nondestructive testing techniques [5], developing a tension monitoring system for cable-stayed bridges using wireless smart sensors and vibration-based tension estimation methods [6], and tension material tension force monitoring in PSC girders and cable-stayed bridges using FBG sensors [7,8]. Moreover, in recent years, tension material monitoring research has expanded to other fields, such as aerospace, mooring lines in subsea structures, and nuclear containment building. Research has been conducted on building a structural health monitoring system for spacecraft segments based on fiber optic sensors and the calculation of the strain response difference characteristics [9]. Studies have been conducted on the dynamic instability behavior of suspended floating tunnel (SFT) using fluid, structural, and dynamic analysis techniques [10] and also on the development of offshore structural health monitoring using fiber bragg grating (FBG) sensors [11]. Research has been conducted to develop a model for reliability analysis considering the tension force loss in the tension materials used in reactor containment buildings [12]. Monitoring technologies used in these various fields enhance the stability of structures and facilitate maintenance, thereby protecting lives and property. However, in environments such as space, oceans, and nuclear reactors, there are limitations to their field applicability, necessitating further research to improve them.

In this study, an elasto-magnetic (E/M) sensor was used to monitor the tension force of a tension member. E/M sensors are a nondestructive measurement method suitable for monitoring tension members in structures, as their characteristics do not change over time and are highly accurate and reliable [13,14,15]. E/M sensors are highly accurate and reliable and have been extensively studied for managing and estimating tension losses in cables and steel rods in structures such as bridges, tunnels, and slopes [16,17,18]. However, the existing magnetization structure, the solenoid-type, has limitations in field usability because the coil must be wound by hand in the field. To overcome this problem, this study improves the magnetization structure of the E/M sensor to a divided yoke-type sensor that is openable. The yoke-type E/M sensor was developed through numerical design and a manufacturing process through simulation. This was applied to a tension measurement device to collect the voltage signal induced in the secondary coil while increasing the tension force in a stepwise manner. The magnitudes of the highest and lowest peak-to-peak voltages of the collected induced voltage signals were indexed and used for analysis. Based on this, the pattern of the induced voltage signal magnitude change according to the change in the tension force of the solenoid and yoke-type E/M sensors was analyzed, and the performance of the yoke-type E/M sensor was verified.

The contents of this paper are organized as follows. Section 2 presents the theoretical background of the E/M sensor. Section 3 deals with the development of the yoke-type E/M sensor, and experiments to measure the tensile stress of tension members based on the developed yoke-type E/M sensor are discussed in Section 4. Section 5 presents the results and analysis of induced voltage signals according to tension changes in the yoke-type E/M sensor. Section 6 is the conclusion and future plans.

## 2. Principle of Elasto-Magnetic Sensor

### 2.1. Reverse Magnetostrictive Effect for Detecting Tension Force Changes

The magnetostrictive effect refers to an alteration in the configuration or size of a magnetic entity due to fluctuations in the surrounding magnetic field [19]. The relationship for the magnetostrictive effect is given by Equation (1). The magnetostrictive effect, represented by λ, denotes strain, which is the ratio between the changed length of the magnet and the original length.

(1) $$\lambda =\frac{∆l}{l}$$

The opposite of the magnetostrictive effect is the reverse magnetostrictive effect, which is a change in the magnetic properties when a magnetic material is stressed. The fundamental principle of estimating the tension force of a tension material using an E/M sensor is based on the reverse magnetostrictive effect.

(2) $$\frac{dl}{l}=\frac{3}{2}\lambda_{\sigma }\left(\cos^{2}\theta -\frac{1}{3}\right)$$

**Figure 1:**
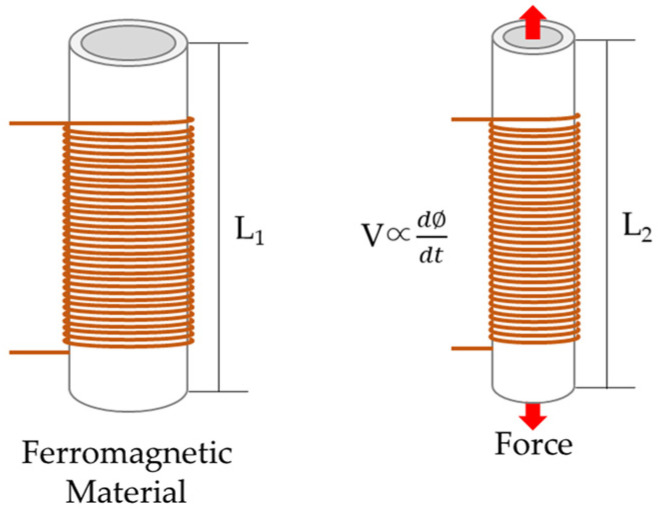
Conceptual diagram of the reverse magnetostrictive effect.

In Equation (2), θ represents the angle between the magnetization effect and the externally applied force, and as shown in Figure 1, when a force is applied from the outside, the magnetic properties change due to the magnetostrictive effect. Therefore, when a tension force is applied to a ferromagnetic body, the magnetic properties change, and the tension force can be measured [20,21,22].

### 2.2. Faraday’s Law of Electromagnetic Induction for Flux Density Measurement

**Figure 2:**
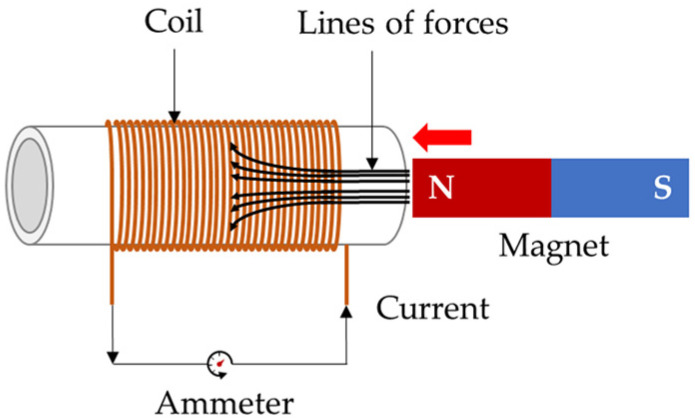
Conceptual diagram of Faraday’s law of electromagnetic induction.

Faraday’s law of electromagnetic induction was proposed by Michael Faraday in 1831 [23]. When a magnetic field exists in free space, a magnetic flux Φ is generated, and when the magnetic flux changes, an electromotive force is induced. This implies that the induced voltage is proportional to the rate of change of the magnetic flux [24,25]. The corresponding formula is expressed in Equation (3), and Faraday’s law of electromagnetic induction is shown in Figure 2.

(3) $$V=-N\frac{d\Phi }{dt},$$

where V is the induced voltage, N is the number of turns of the coil, and $\frac{d\Phi }{dt}$ is the rate of change of the magnetic flux over time. Magnetic induction B is the flux density, which is equal to Equation (4).

(4) $$B=\frac{\Phi }{A},$$

where B is the magnetic induction density representing the strength of the magnetic field, Φ is the magnetic flux, and A is the surface area through which the magnetic field passes. Substituting Equation (4) into the electromagnetic induction law gives us Equation (5).

(5) $$V=-NA\frac{dB}{dt}$$

As a change in the flux density indicates a change in the physical properties of the specimen, a change in the flux signal was detected when a tension force was applied. Thus, the flux density of the magnetized ferromagnet was measured.

### 2.3. Principle of Solenoid-Type E/M Sensor

**Figure 3:**
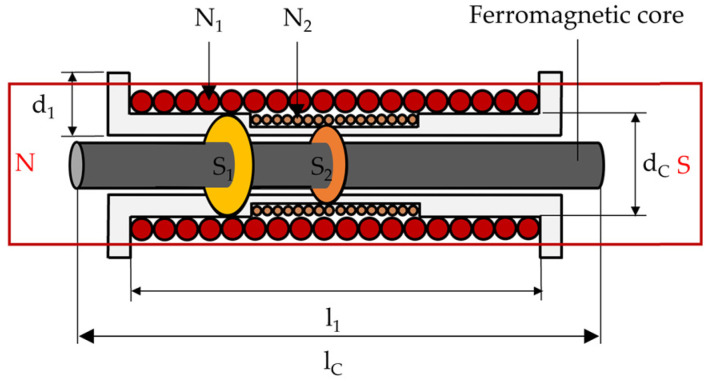
Schematic of solenoid-type E/M sensor.

The solenoid-type E/M sensor is composed of a primary coil that generates a magnetic field and a secondary coil that measures the generated magnetic field, as shown in Figure 3. The principle of the solenoid-type E/M sensor is that a strong magnetic field is formed around the specimen inside the coil when an alternating current is applied to the primary coil, and the specimen is magnetized. The flux densities of the magnetized specimens were measured using Faraday’s law of electromagnetic induction.

### 2.4. Principle of Yoke-Type E/M Sensor

**Figure 4:**
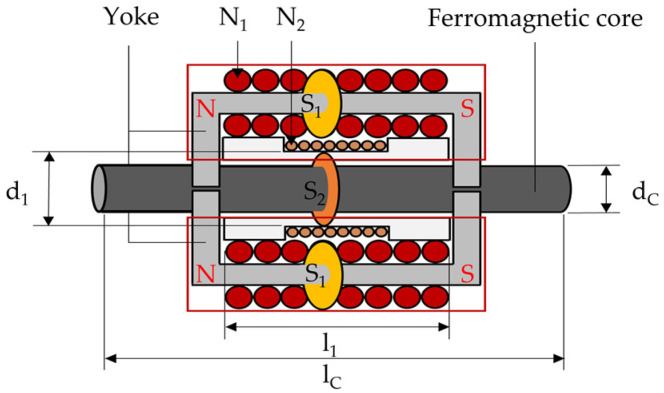
Schematic of yoke-type E/M sensor.

The yoke-type E/M sensor developed to improve field usability is a ferromagnetic yoke, as shown in Figure 4, in which the primary coil magnetizes the yoke, and the magnetized yoke acts as a magnet by forming N and S poles. When these two yokes combine with the specimen, they form a closed loop, indirectly magnetizing the specimen and creating a magnetic field.

Regarding the notation used in Figure 3 and Figure 4, N is the number of turns in the coil, S is the internal cross-sectional area of the coil, d is the diameter of the coil, and l is the length of the coil.

### 2.5. Wireless Communication Data Collection Equipment for E/M Sensor Signal Acquisition

**Figure 5:**
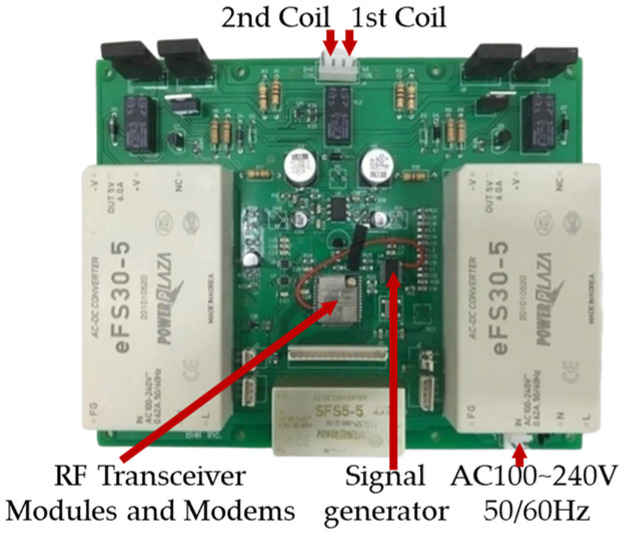
Wireless communication data collection equipment specification for E/M sensor signal acquisition.

The wireless communication data acquisition device (DAQ) operates in WIFI AP mode in conjunction with a smartphone. The voltage supplied to the primary coil of the E/M sensor connected to the DAQ was measured 500 times at 0.004 s intervals for 2 s. These values were used as *y*-axis values, and the *x*-axis was set at a time interval of 0.004 s (Figure 5).

**Figure 6:**
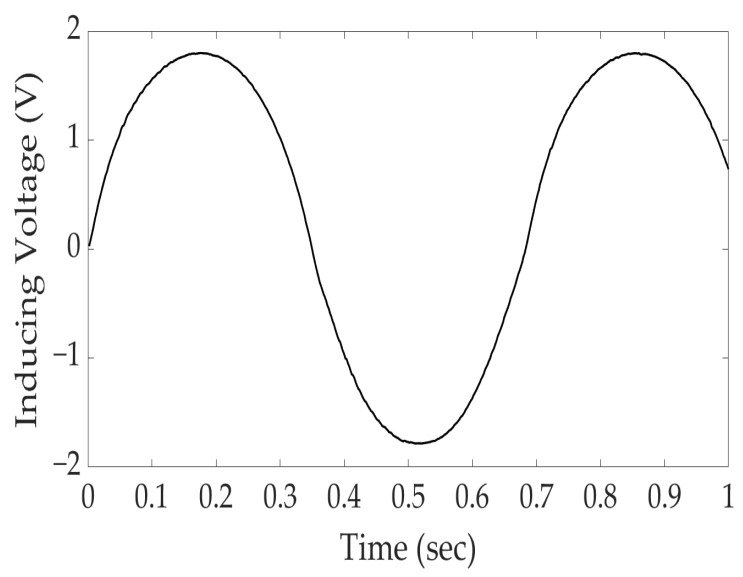
Primary coil excitation voltage signal sample.

The E/M sensor supply signal connected to the DAQ was a sine-shaped signal with a period of 0.4755 and an amplitude of 1.7202 V, as shown in Figure 6. In this method, a voltage signal is applied to the primary coil, and the voltage signal induced by the secondary coil is measured. This can be utilized for damage detection and monitoring of the specimens.

### 2.6. Derivation of Peak-to-Peak Value for Quantification of Induced Voltage Signal

**Figure 7:**
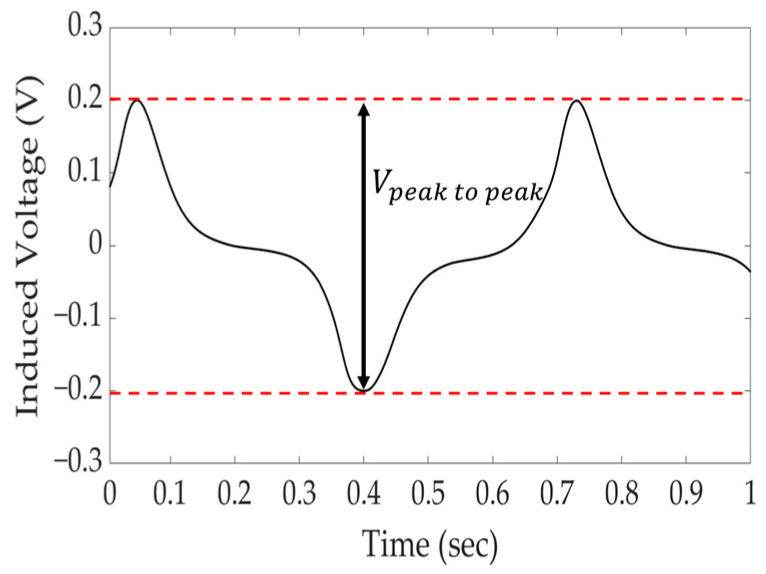
Secondary coil-induced voltage signal sample and peak-to-peak value derivation for signal quantification.

To estimate the tension force, the peak-to-peak value of induced voltage, which comprised the highest and lowest voltages of the induced voltage signal measured for each ton, was derived by exponentiating [26]. The derived peak-to-peak value of induced voltage was analyzed and compared with those of the solenoid- and yoke-type E/M sensors in Section 5 to verify the reliability of the yoke-type E/M sensor (Figure 7).

## 3. Development of Yoke-Type E/M Sensor

### 3.1. Simulation for Numerical Design of E/M Sensor

**Figure 8:**
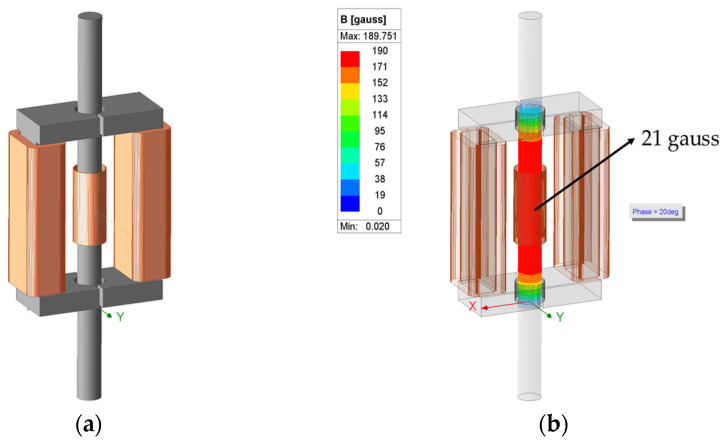
Numerical design for yoke-type E/M sensor simulation results; (**a**) 3D modeling and (**b**) magnetic flux measurement results of yoke-type E/M sensor using eddy current solution-type simulation.

In this study, the ANSYS Maxwell software 2023 R1 was used for the numerical design of the yoke-type E/M sensor. This software is based on Maxwell’s equations and performs electromagnetic finite element (FE) simulations. It performs advanced simulations and analyses of electromagnetic field problems and is commonly used in various fields [27,28]. Using ANSYS Maxwell, 3D modeling of the yoke-type E/M was performed by setting conditions such as the diameter, voltage, resistance, inductance, number of turns, winding length, and number of layers of the primary and secondary coils, as shown in Table 1. The yoke was designed as a ferromagnet made of carbon steel with a width of 34 mm, a length of 30 mm, a height of 100 mm, and a circular radius of 8 mm. The specimen was a PC steel wire with a diameter of Φ12.7 mm and a length of 3 m. The yoke and specimen were made of steel 1008, and the coil was made of copper. The modeling was performed as shown in Figure 8a.

After 3D modeling of the sensor and specimen, the magnetic flux was measured by simulating the measurement of a real yoke-type E/M sensor based on the ANSYS Maxwell eddy current solution [29]. Consequently, the magnetic flux density at the center of the specimen was measured at 21 gauss, as shown in Figure 8b, and a yoke-type E/M sensor was fabricated based on the simulation. As the magnetic field strength of a sensor can vary depending on the core type, winding diameter, number of turns, and input current magnitude, the internal and external conditions of the sensor must be set appropriately.

### 3.2. Fabrication of Yoke-Type E/M Sensor for Measuring Tension Force

**Figure 9:**
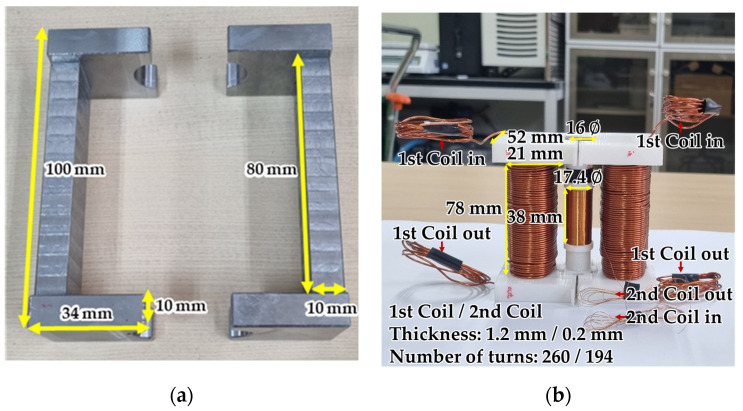
Fabrication of yoke-type E/M sensor; (**a**) yoke and (**b**) yoke-type E/M sensor.

A yoke-type E/M sensor head is constructed using a retractable case. The primary coil wound on each yoke was 1.2 mm thick and wound 260 times, and the secondary coil in the inner cylindrical part was 0.2 mm thick and wound 194 times. In this case, the yoke was 34 mm wide, 30 mm long, and 100 mm high, with a circular radius of 8 mm, as shown in Figure 9a. The sensor was fabricated as shown in Figure 9b.

### 3.3. Magnetic Field Measurement of Solenoid-Type and Yoke-Type Sensors Using a Gaussmeter

**Figure 10:**
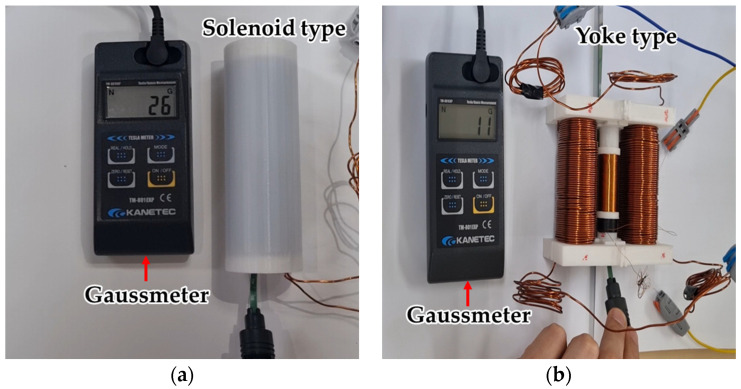
Magnetic field measurement using a gaussmeter; (**a**) solenoid-type E/M sensor and (**b**) yoke-type E/M sensor.

As a result of measuring the magnetic flux density at the center of the solenoid and yoke-type E/M sensors using a gaussmeter, as shown in Figure 10. It was found to be approximately 26 G (0.0026 T) for the solenoid-type E/M sensor. The yoke-type E/M sensor was measured at 11 G (0.0011 T), which was 40.54% lower than that of the solenoid-type E/M sensor. However, in contrast to the solenoid-type, which must be wired directly into the field, the yoke-type can be opened or closed. Therefore, it is superior in terms of field usability.

(6) $$B=\mu_{0}H,\;H=\frac{N\cdot I}{L}$$

To increase the magnetic field strength of the yoke-type E/M sensor, a method of increasing the magnetic field strength using Ampere’s law will be considered in future research [30,31]. Ampere’s law can be expressed using Equation (6), where B is the magnetic flux density, $\mu_{0}$ is the vacuum permeability, H is the magnetic field strength around the coil (solenoid), N is the number of turns in the coil, and I is the current applied to the coil. According to Ampere’s law, the magnetic field strength of a sensor can be increased by increasing the number of turns in the coil [24].

## 4. Experimental Studies for Tension Force Estimation Using E/M Sensor

### Experimental Setup and Progress for Tension Force Measurement

**Figure 11:**
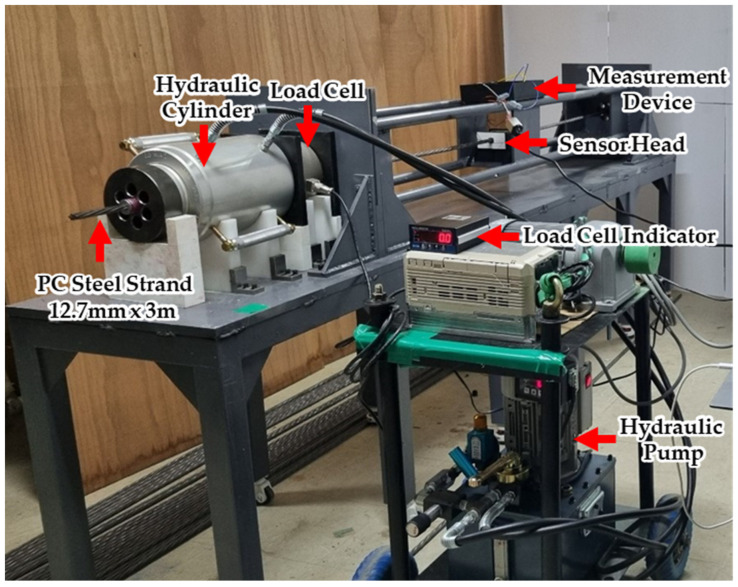
Experimental setup for tension force measurement.

In this study, an experimental device was configured to estimate the tension of the PS tendons, as shown in the figure below. The specimen was a PC steel wire with a length of 3 m and a diameter of 12.7 mm, and a tension reloader consisting of a PC steel wire, a load cell, and a hydraulic cylinder was used, as shown in Figure 11. A PC steel wire was installed such that it could pass through the sensor head, and the experimental apparatus was constructed by connecting the instrument and sensor head to apply and induce a signal to process the measured signal.

**Figure 12:**
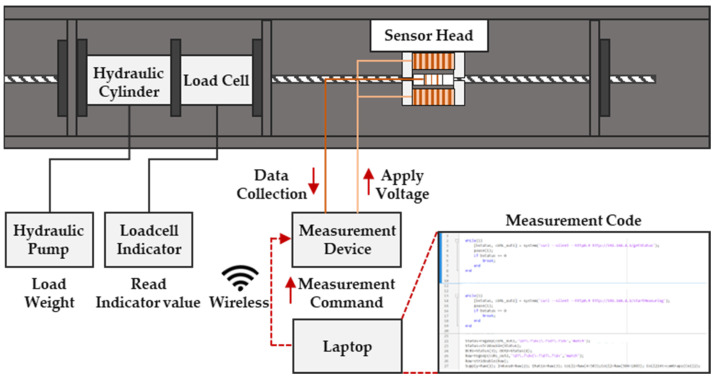
Tension force experimental flowchart.

In the tension measurement experiment, the load was increased from 0 to 10 tons at 1-ton intervals using a hydraulic cylinder and pump and measured 10 times at each stage based on the load cell indicator. This process was repeated ten times. The applied and induced voltage signals from the yoke-type E/M sensor, measured using this setup, were transmitted to the controller via a wireless communication DAQ. The transmitted signal data were processed and analyzed using a laptop (Figure 12).

## 5. Experimental Results and Analysis

### 5.1. The Induced Voltage Signal of the Secondary Coil with Increasing Tension Results (Solenoid-Type E/M Sensor)

**Figure 13:**
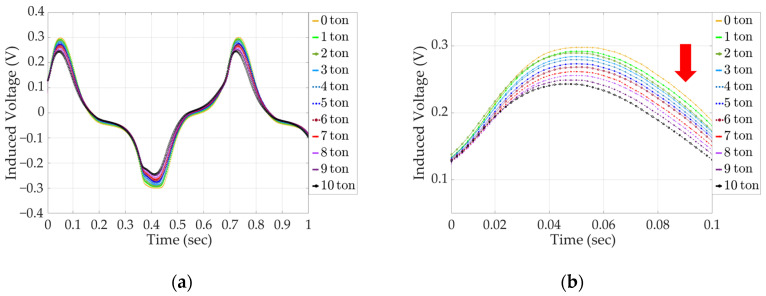
Induced voltage signal with increasing tension in a solenoid-type E/M sensor; (**a**) full signal data and (**b**) enlarged signal data.

**Figure 14:**
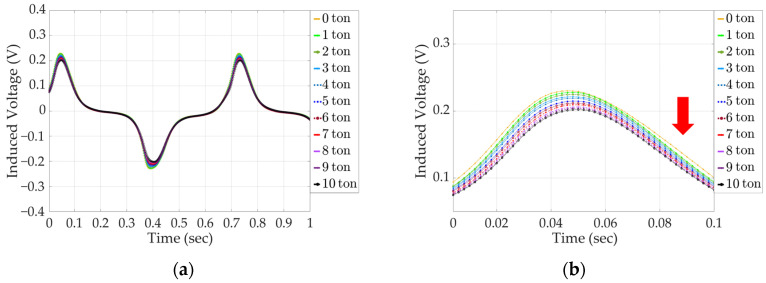
Induced voltage signal with increasing tension in a yoke-type E/M sensor; (**a**) full signal data and (**b**) enlarged signal data.

The results of the analysis of the secondary coil-induced voltage signals of the solenoid and yoke-type E/M sensors as the tension force increases from 0 to 10 tons are shown in Figure 13, and an enlarged signal data graph of the first peak, from 0 to 0.1 s, is shown in Figure 14. For the solenoid-type E/M sensor, the maximum voltage at 0 tons is 0.3 V, and the maximum voltage at 10 tons is 0.24 V, which is a difference of 0.06 V.

### 5.2. The Induced Voltage Signal of the Secondary Coil with Increasing Tension Results (Yoke-Type E/M Sensor)

For the yoke-type E/M sensor, the maximum voltage at 0 tons is 0.23 V, the maximum voltage at 10 tons is 0.2 V, and the difference between the maximum voltage at 0 tons and 10 tons is 0.003 V. The difference in the maximum induced voltage owing to the increase in the tension force of the yoke-type E/M sensor compared to that of the solenoid-type E/M sensor was 50%. However, the same pattern of a gradual decrease in the maximum value of the signal for each ton was observed. This confirms that the yoke-type E/M sensor can estimate the tension force of tension materials as well as the solenoid-type E/M sensor.

### 5.3. Peak-to-Peak Results with Increasing Tension (Solenoid-Type E/M Sensor)

**Figure 15:**
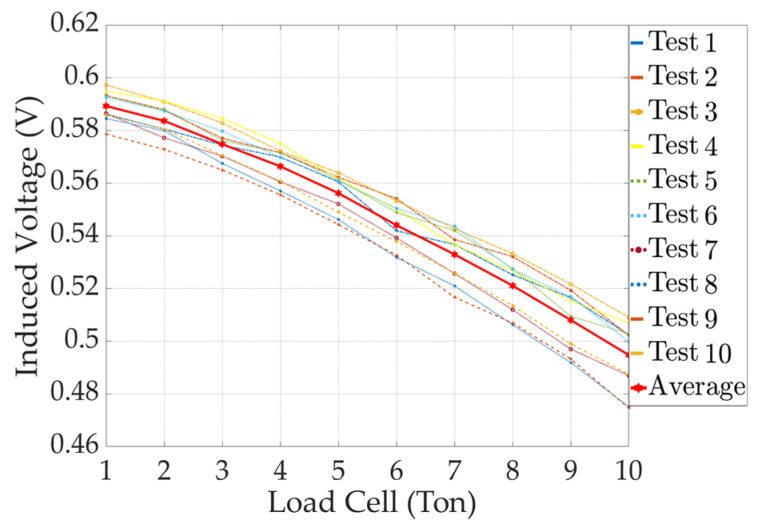
Peak-to-peak results with increasing tension in a solenoid-type E/M sensor.

In this study, the peak-to-peak index was used for signal processing, which offers the advantages of simplified data processing and noise robustness. Utilizing this index, the peak-to-peak value of induced voltages of a total of 10 tests of the solenoid-type and yoke-type E/M sensors were derived according to the tension. A comparative analysis was performed based on the derived data, and the reliability of the yoke-type E/M sensor was verified. A peak-to-peak graph of the solenoid-type E/M sensor is shown in Figure 15.

By analyzing the peak-to-peak graph for the solenoid-type E/M sensor, we observed a decreasing peak-to-peak pattern as the tension increased with each iteration.

### 5.4. Peak-to-Peak Results with Increasing Tension (Yoke-Type E/M Sensor)

**Figure 16:**
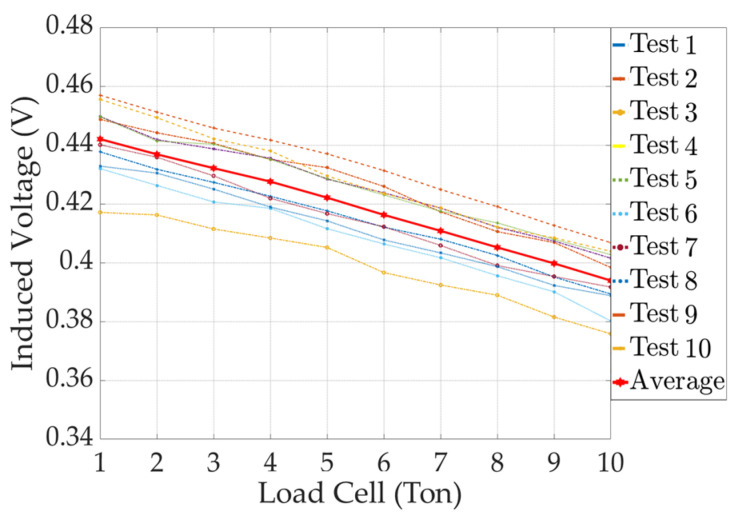
Peak-to-peak results with increasing tension in a yoke-type E/M sensor.

A peak-to-peak graph of the yoke-type E/M sensor is shown in Figure 16.

Although the magnitude of the error in the peak-to-peak graph for the yoke-type E/M sensor was larger than that for the solenoid-type E/M sensor, we observed the same pattern of the graph shifting downward as the tension increased. In the following section, a linear regression analysis is performed on the peak-to-peak average values of the solenoid and yoke-type E/M sensors to derive an output formula for tension estimation.

### 5.5. The Linear Regression Analysis Results of Peak-to-Peak Value of Induced Voltages (Solenoid-Type E/M Sensor)

**Figure 17:**
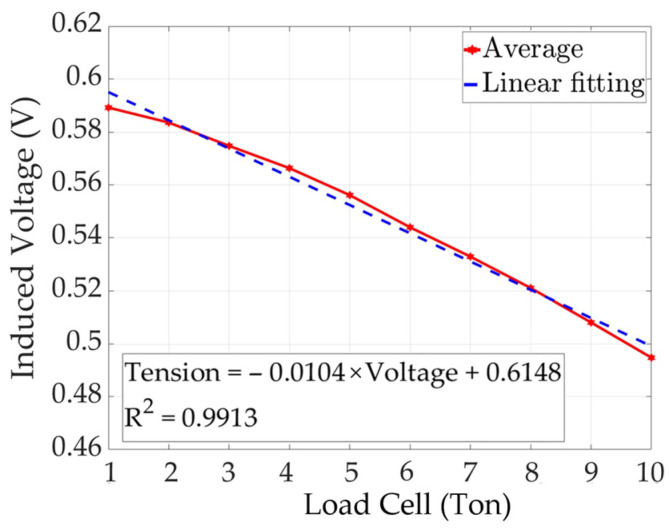
Peak-to-peak linear fitting of solenoid-type E/M sensor.

The results of linear regression analysis of the peak-to-peak average value of the solenoid-type E/M sensor are shown in Figure 17.

(7) $$Tension\left(ton\right)=-0.0104\times Voltage+0.6148$$

Linear regression analysis of the peak-to-peak average value of the solenoid-type E/M sensor showed a coefficient of determination (R-squared) of 0.9913. The formula for calculating tension is given by Equation (7). The induced voltage signal and tension are proportional to each other and can be used to estimate the tension of the solenoid-type E/M sensor.

### 5.6. The Linear Regression Analysis Results of Peak-to-Peak Value of Induced Voltages (Yoke-Type E/M Sensor)

**Figure 18:**
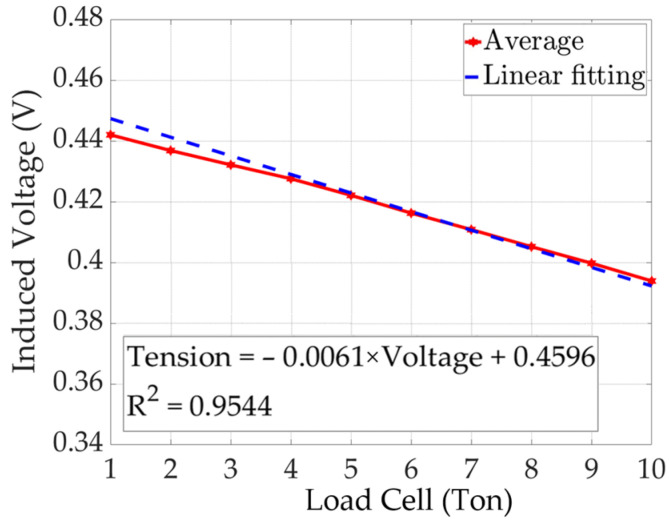
Peak-to-peak linear fitting of yoke-type E/M sensor.

The results of the linear regression analysis of the peak-to-peak average values of the yoke-type E/M sensor are shown in Figure 18.

(8) $$Tension\left(ton\right)=-0.0061\times Voltage+0.4596$$

Linear regression analysis of the peak-to-peak average values of the yoke-type E/M sensor showed a coefficient of determination (R-squared) of 0.9544. The formula for calculating the tension is expressed as Equation (8). The induced voltage signal and tension are proportional to each other and can be used to estimate the tension of the yoke-type E/M sensor.

Linear regression analysis of the average peak-to-peak values of the solenoid- and yoke-type E/M sensors confirmed that both graphs show the same downward pattern. Based on these analyses, we believe that the yoke-type E/M sensor proposed in this study has sufficiently improved field usability and performance through performance verification of the solenoid-type E/M sensor.

## 6. Conclusions

An experimental study was conducted to verify the applicability of the E/M sensors for estimating the tension force of the tension members. The solenoid-type E/M sensor, which is the magnetization structure of conventional E/M sensors, is difficult to apply to continuum specimens in the field because of its closed-loop form, and there are limitations in field usability that require winding the coil directly in the field. In this paper, we proposed a divided yoke-type E/M sensor that can be operated.

The ANSYS Maxwell software 2023 R1 was used for the numerical design of the yoke-type E/M sensor. By setting the internal and external conditions of the sensor, specimen, and yoke, it was confirmed that a magnetic flux was formed at the center of the sensor and specimen in the simulation based on the eddy current solution. Accordingly, a yoke-type E/M sensor was fabricated. The magnetic flux density measured at the center using a gaussmeter was found to be lower than that of the solenoid-type sensor. However, according to Ampere’s law, increasing the number of turns in the coil is expected to sufficiently increase the magnetic flux. The fabricated sensor was applied to a tension-testing apparatus, where the load was increased from 0 to 10 tons at intervals of 1 ton. At each stage, measurements were performed 10 times using a load cell indicator, and this process was repeated over 10 experiments. The measured applied and induced voltage signals of the yoke-type E/M sensor were transmitted to the controller via a wireless communication DAQ. The transmitted signal data were processed and analyzed using a laptop.

After analyzing the signal of the yoke-type E/M sensor, it was found that the voltage signal that was induced as the tension force increased was half that of the solenoid-type E/M sensor. However, the pattern of a decreasing maximum signal value for each ton was the same as that of the solenoid-type. In addition, the peak-to-peak value of the induced voltage signal was derived and analyzed, and it was confirmed that the graph exhibited the same pattern as that of the solenoid-type signal as the tension force increased. The average peak-to-peak value was used to derive the tension force formula for the solenoid- and yoke-type E/M sensors through linear regression. Based on the results of this analysis, we believe that the yoke-type E/M sensor has been sufficiently verified to improve usability and performance in the field. Therefore, it was confirmed that the tension force of a tension member can be estimated using the proposed yoke-type E/M sensor. This is expected to become an effective tension monitoring technology through performance optimization and usability verification studies for each target tension member in the future.

## Figures and Tables

**Table 1:** Setting conditions for coil in yoke-type E/M sensor simulation.

Coil	Material	Diameter (mm)	Voltage (V)	Resistance (Ohm)	Inductance	Number of Turns	Winding Length (mm)	Number of Layers
1st	Copper	1.2	1.75	10	3372.7 μH	260	15.6	4
2nd		0.2	0	0.36	531.13 μH	194	7.6	2

## Data Availability

Data are contained within the article.

## Abbreviations & Nomenclature

The following abbreviations and Nomenclatures are used in this manuscript:

PSC	Prestressed Concrete
MFL	Magnetic Flux Leakage
FBG	Fiber Bragg Grating
E/M	Elasto Magnetic
SFT	Suspended Floating Tunnel
DAQ	Data Acquisition
FE	Finite Element
B	Magnetic flux density [T]
H	Magnetic field intensity [A/m]
λ	Strain
N	Number of turns in the coil [Turn]
S	Internal cross-sectional area of the coil [$m^{2}$]
d	Diameter of the coil [m]
l	Length of the coil [m]
Φ	Magnetic flux [T·$m^{2}$]
$\mu_{0}$	Vacuum permeability [kg·m·s/$A^{2}$]
I	Current applied to the coil [A]

## References

[1] Wang J.H.-C. (2006). Mechanobiology of tendon. J. Biomech..

[2] Daniele M., Nicola S., Andrea O., Rui P., Matteo M., Gian M. (2020). Numerical study on the collapse of the Morandi bridge. J. Perform. Constr. Facil..

[3] Hong J., Chiew Y., Lu J., Lai J., Lin Y. (2012). Houfeng bridge failure in Taiwan. J. Hydraul. Eng..

[4] Kim I., Jung H., Yoon S., Park J. (2023). Dynamic Response Measurement and Cable Tension Estimation Using an Unmanned Aerial Vehicle. Remote Sens..

[5] Kim J., Choi J., Lee E., Park S. (2014). Field Application of a Cable NDT System for Cable-Stayed Bridge Using MFL Sensors Integrated Climbing Robot. J. Korean Soc. Nondestruct. Test..

[6] Sim S., Li J., Park J., Cho S., Spencer J.R., Jung H. (2014). A wireless smart sensor network for automated monitoring of cable tension. Smart Mater. Struct..

[7] Yao Y., Yan Y., Bao Y. (2021). Measurement of cable forces for automated monitoring of engineering structures using fiber optic sensors: A review. Autom. Constr..

[8] Kim J., Kim H., Park Y., Yang I., Kim Y. (2012). FBG sensors encapsulated into 7-wire steel strand for tension monitoring of a prestressing tendon. Adv. Struct. Eng..

[9] Xu J., Zeng J., Chen B., Lu R., Zhu Y., Qi L., Chen X. (2023). Spacecraft Segment Damage Identification Method Based on Fiber Optic Strain Difference Field Reconstruction and Norm Calculation. Sensors.

[10] Thies P., Johanning L., Harnois V., Smith H., Parish D. (2014). Mooring line fatigue damage evaluation for floating marine energy converters: Field measurements and prediction. Renew. Energy.

[11] Murawski L., Ostachowicz W., Opoka S., Mieloszyk M., Majewska K. (2012). Practical application of monitoring system based on optical sensors for marine constructions. Key Eng. Mater..

[12] Kim S., Choi J., Joung Y. (2013). Long-term reliability evaluation of nuclear containments with tendon force degradation. Nucl. Eng. Des..

[13] Jarosevic A. (1999). Magnetoelastic Method of Stress Measurement in Steel. Smart Struct..

[14] Wang M., Chen Z., Koontz S., Lloyd G. (2000). Magnetoelastic permeability measurement for stress monitoring in steel tendons and cables. Proc. SPIE-Int. Soc. Opt. Eng..

[15] Sumitro S., Kurokawa S., Shimano K., Wang M. (2005). Monitoring based maintenance utilizing actual stress sensory technology. Smart Mater. Struct..

[16] Wang M., Wang G., Zhao Y. (2005). Application of EM stress sensors in large steel cables. Sensing Issues Civil Struct Health Monitoring.

[17] Kim W., Kim J., Park J., Kim W., Park S. (2022). Verification of tensile force estimation method for temporary steel rods of FCM bridges based on area of magnetic hysteresis curve using embedded elasto-magnetic sensor. Sensors.

[18] Kim J., Kim J., Lee C., Park S. (2017). Development of embedded EM sensors for estimating tensile forces of PSC girder bridges. Sensors.

[19] Joule J. (1842). On the electric origin of the heat of combustion. Philos. Mag..

[20] Lee E. (1955). Magnetostriction and magnetomechanical effects. Rep. Prog. Phys..

[21] Lee E. (1958). Magnetostriction curves of polycrystalline ferromagnetics. Proc. Phys. Soc..

[22] Belahcen A., Peusssa T., Singh D., Rasilo P. Computation of the inverse magnetostriction and its application in mechanical stress sensing. Proceedings of the 9th IET International Conference on Computation in Electromagnetics (CEM 2014).

[23] Davino D., Giustiniani A., Visone C. (2010). Design and test of a stress-dependent compensator for magnetostrictive actuators. IEEE Trans. Magnet..

[24] Jiles D. (2015). Introduction to Magnetism and Magnetic Materials.

[25] Zuza K., Guisasola J., Michelini M., Santi L. (2012). Rethinking Faraday’s law for teaching motional electromotive force. Eur. J. Phys..

[26] Ha S., Park S., Lim H., Baek S., Kim D., Yoon S. (2019). Analysis of the Optimal Location of Wearable Biosensor Arrays for Individual Combat System Considering Both Monitoring Accuracy and Operational Robustness. J. Korea Inst. Mil. Sci. Technol..

[27] Park J., Oh J., Kim Y. (2022). Design and Control of Multi-Plate MR Clutch Featuring Friction and Magnetic Field Control Modes. Sensors.

[28] Suresh A., Abudhahir A., Daniel J. (2017). Development of magnetic flux leakage measuring system for detection of defect in small diameter steam generator tube. Measurement.

[29] Ko D., Park J., Kim J., Lee C., Yoon H., Park S. (2023). Experimental Study for Nondestructive Evaluation of Embedded Tendons in Ground Anchors Using an Elasto-Magnetic Sensor: Verification Through Numerical Finite Element Simulations. IEEE Sens. J..

[30] Al-Shaikhli T., Ahmad B., Al-Taweel M. (2019). The implementations and applications of ampere’s law to the theory of electromagnetic fields. Int. J. Adv. Sci. Technol..

[31] Puaypung W., Rakkapao S. (2018). A low-cost Arduino microcontroller for measuring magnetic fields in a solenoid. J. Phys. Conf. Ser..

